# LncRNA7503 decreases peach (*Prunus persica*) branch number and angle by inducing pre-miR395a degradation and reducing bioactive BR content

**DOI:** 10.1186/s43897-025-00215-6

**Published:** 2026-05-07

**Authors:** Xiaobei Wang, Hefang Xie, Wan Pei, Ruixian Shen, Yajia Zhang, Junjie Zhang, Xiaodong Lian, Haipeng Zhang, Xianbo Zheng, Nan Hou, Lei Wang, Jun Cheng, Wei Wang, Langlang Zhang, Xia Ye, Jidong Li, Jiancan Feng, Bin Tan

**Affiliations:** 1 College of Horticulture, Henan Agricultural University, 218 Pingan Road, Zhengzhou, 450046 China; 2 Henan Engineering and Technology Center for Peach Germplasm Innovation and Utilization, Zhengzhou, 450046 China; 3 International Joint Laboratory of Henan Horticultural Crop Biology, 218 Pingan Road, Zhengzhou, 450046 China; 4 College of Forestry, Henan Agricultural University, 218 Pingan Road, Zhengzhou, 450046 China

**Keywords:** Peach, Branch number/angle, Brassinosteroid metabolism, *PpCYP734A14*, MiRNA, LncRNA

## Abstract

Peach (*Prunus persica* L.) branch number and angle significantly influence orchard management efficiency, fruit yield, and quality. However, few reports exist on genes regulating both traits characteristics. This study utilized miRNA sequencing to identify *PpCYP734A14*, one of four BR pathway-related genes targeted by differentially expressed miRNAs, as a participant in BR metabolism with elevated expression in pillar-type peach cultivar Zhaoshouhong (ZSH). Transient expression analyses confirmed that miR395a-3p targets *PpCYP734A14*. The overexpression of pre-miR395a led to significant reduction in *PpCYP734A14* mRNA levels and increased bioactive BR content, resulting in enhanced branching and branch angle. Conversely, miR395a-3p silencing produced opposite effects. LncRNA-miRNA-mRNA network analysis revealed that differentially expressed lncRNA7503 reduced mature miR395a-3p levels by promoting the posttranscriptional degradation of pre-miR395a, subsequently increasing *PpCYP734A14* expression. These findings implicate *PpCYP734A14* in the regulation of both branch number and angle through a lncRNA7503-miR395a-3p mechanism. This pathway presents valuable molecular tools for genetic improvement of tree architecture and facilitates labor-saving cultivation approaches.

**Supplementary Information:**

The online version contains supplementary material available at 10.1186/s43897-025-00215-6.

## Core

Branch number and angle represent critical agronomic traits that determine tree architecture. Research has demonstrated that miR395a-3p influences peach endogenous bioactive BR content through targeting *PpCYP734A14*, which converts active BR to its inactive form. Additionally, lncRNA7503 substantially enhanced *PpCYP734A14* expression by promoting pre-miR395a degradation. The expression levels of *PpCYP734A14* and miR395a-3p have significant effects on branch number and angle, establishing that the lncRNA7503-miR395a-3p pathway modulates peach branch formation through BR metabolism regulation.

## Gene & accession numbers

Gene sequences from this study are accessible in the Phytozome database (Phytozome (doe.gov) under accession: PpCYP734A14 (Prupe.1G296900)

## Introduction

Shoot architecture, encompassing branch number, branch angle, and node length, serves a fundamental role in determining tree architecture and directly influences fruit yield and quality (Wang et al. 2018; Cheng et al. 2019; Duan et al. 2025). Peach ranks as the fourth-largest deciduous fruit crop and maintains widespread consumer popularity. Peach tree architecture encompasses several classifications, including standard, dwarf, pillar, compact, weeping, and spur types (Bassi et al. 1994). The standard type predominates in cultivation despite its tendency toward excessive vegetative growth. The pillar type, characterized by reduced branching and narrower branch angles, offers significant advantages in reducing pruning requirements and orchard management costs (Bassi et al. 1994; Kotov et al. 2021).

Branch number and angle formation are influenced by various environmental factors and internal signals (Wang et al. 2020; Kotov et al. 2021; Cao et al. 2022). Research has traditionally focused on examining branch number or angle independently (Dou et al., 2022; Kotov et al. 2021; Zhang et al. 2025). These characteristics, which determine canopy dimensions, involve multiple developmental stages including axillary meristem initiation, lateral bud formation, bud activation, and sustained branch development (Wang et al. 2018). Auxin functions as a primary hormone that suppresses bud outgrowth through apical dominance maintenance. However, auxin's influence on shoot branching operates indirectly through strigolactones (SLs). Alterations in the SL pathway substantially affect branching, with the SL-responsive gene inhibiting BRANCHED1 (BRC1)/TB1 serving as a critical regulator of bud outgrowth (Kotov et al. 2021).

Branch angle, which determines branch growth direction, is primarily regulated by auxin. The genes *LAZY1* and TILLER ANGLE CONTROL 1 (TAC1) control shoot gravitropism by regulating endogenous IAA distribution (Li et al. 2021; Liu et al. 2024). IAA exhibits negative regulation of branch angle formation, and alterations in auxin-related genes significantly influence leaf and branch angles (Chen et al. 2018; Feng et al. 2023). In contrast, brassinosteroids (BRs) demonstrate positive regulation of branch angle, with BR biosynthesis or signaling gene mutations resulting in erect leaf phenotypes in rice (Xia et al. 2015; Liu et al. 2019).

BRs participate in the regulation of both cell expansion and branch formation (Xia et al. 2021; Wang et al. 2024). Unlike auxin, BRs lack long-distance transport capability, and their local concentrations depend on synthesis and inactivation rates (Symons and Reid 2004). Cytochrome P450 enzymes, including CYP724B, CYP90A, CYP90B, CYP90D, CYP734A, CYP85A, and CYP90C subfamilies, perform essential functions in BR biosynthesis (Thornton et al. 2011; Toshiyuki et al. 2012; Zhang et al. 2018). Additionally, CYP734A enzymes facilitate BR inactivation by converting bioactive forms such as brassinolide (BL) and castasterone (CS) into inactive forms via C-26 hydroxylation (Ohnishi et al. 2006). Enhanced expression of *CYP734A* genes in tomato and rice induces BR deficiency and dwarf phenotypes through BR inactivation (Ohnishi et al. 2006; Sakamoto et al. 2011). Previous research demonstrated that pillar-type peach exhibits reduced BR synthesis due to decreased expression of *PpD2* (CYP90D1), a key BR biosynthesis gene (Wang et al. 2024). The potential differences in BR inactivation rates between standard and pillar peach, and their contribution to branch number and angle variations, remain to be elucidated.

MicroRNAs (miRNAs), which are approximately 21 nt non-coding RNAs, serve essential regulatory functions in numerous biological processes, including cell fate determination, signal transduction, organ differentiation, and stress response (Bartel 2004; Xu et al. 2024; Zheng et al. 2023). These miRNAs regulate gene expression through translation inhibition or target mRNA cleavage (Pu et al. 2019). For example, miR319 controls rice tillering through suppression of *OsTCP21* (Wang et al. 2021), while miR408-5p modulates auxin signaling through translation repression rather than mRNA cleavage under elevated auxin conditions (Rong et al. 2024).

Recent studies have identified numerous long non-coding RNAs (lncRNAs) as miRNA target mimics (*eTMs*) (Wu et al. 2013; Imaduwage and Hewadikaram 2024), which effectively inhibit miRNA activity. For instance, *MLNC3.2* and *MLNC4.6* enhance anthocyanin accumulation in apple by downregulating miR156a, thereby increasing *SPL* gene expression (Yang et al. 2019). Nevertheless, the precise mechanisms through which lncRNAs inhibit miRNA levels remain to be elucidated.

Our previous study demonstrated that miR6288b-3p regulates peach branching by modulating BR biosynthesis (Wang et al. 2024). To examine whether miRNAs influence BR metabolism and affect branching, we analyzed miRNA sequencing data. Kyoto Encyclopedia of Genes and Genomes (KEGG) analysis of differentially expressed miRNAs (DEMs) identified four BR-related genes, with *PpCYP734A14* being the sole member of the *CYP734A* subfamily involved in BR metabolism. Transient expression assays revealed that miR395a-3p affects bioactive BR levels by targeting *PpCYP734A14*, and transient expression assay in peach seedlings indicated that miR395a-3p could significantly promote peach branching, while *PpCYP734A14* inhibit peach branching. Furthermore, lncRNA7503 was found to regulate *PpCYP734A14* expression by interacting with and degrading pre-miR395a, thereby preventing miR395a-3p from cleaving *PpCYP734A14* mRNA. This investigation seeks to elucidate the potential regulatory mechanisms of the lncRNA7503-miR395a-3p network in controlling peach branch number and angle through regulation of *PpCYP734A14* expression.

## Results

### Pillar peach exhibited smaller branch angle, and exogenous BR treatment significantly increased branch angle

**Fig. 1:**
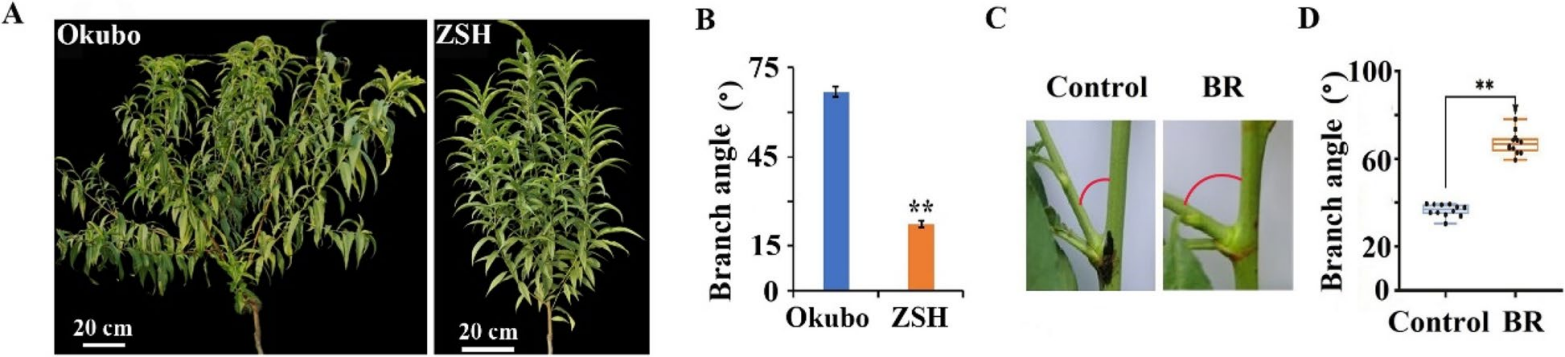
Phenotypic analysis of standard peach Okubo, pillar peach ZSH, and exogenous BR treatment. **A** One-year-old trees of Okubo and ZSH. **B** Branch angles of Okubo were significantly larger than those of ZSH. **C**, **D** Exogenous BR treatment markedly increased peach branch angles compared to the control

To evaluate differences in branch angle between the pillar type peach Zhaoshouhong (ZSH) and the standard type peach Okubo, branch angles of one-year-old trees from both cultivars were measured. The analysis indicated that the branch angle of pillar peach ZSH was significantly smaller than that of standard peach Okubo (Fig. 1A, B). Previous studies also demonstrated that the number of secondary branches in ZSH was significantly lower than in Okubo, and the endogenous BR content in ZSH was markedly reduced. Exogenous BR treatment significantly promoted branching in peach (Wang et al. 2023, 2024).

To validate the role of BR in peach branch angles, synthetic BR was exogenously applied to one-year-old trees of ZSH. Compared to the control group, BR treatment significantly increased the branch angle (Fig. 1C, D). These observations suggest that BR regulates both branch number and branch angle in peach.

### miR395a-3p targets *PpCYP734A14*

**Fig. 2:**
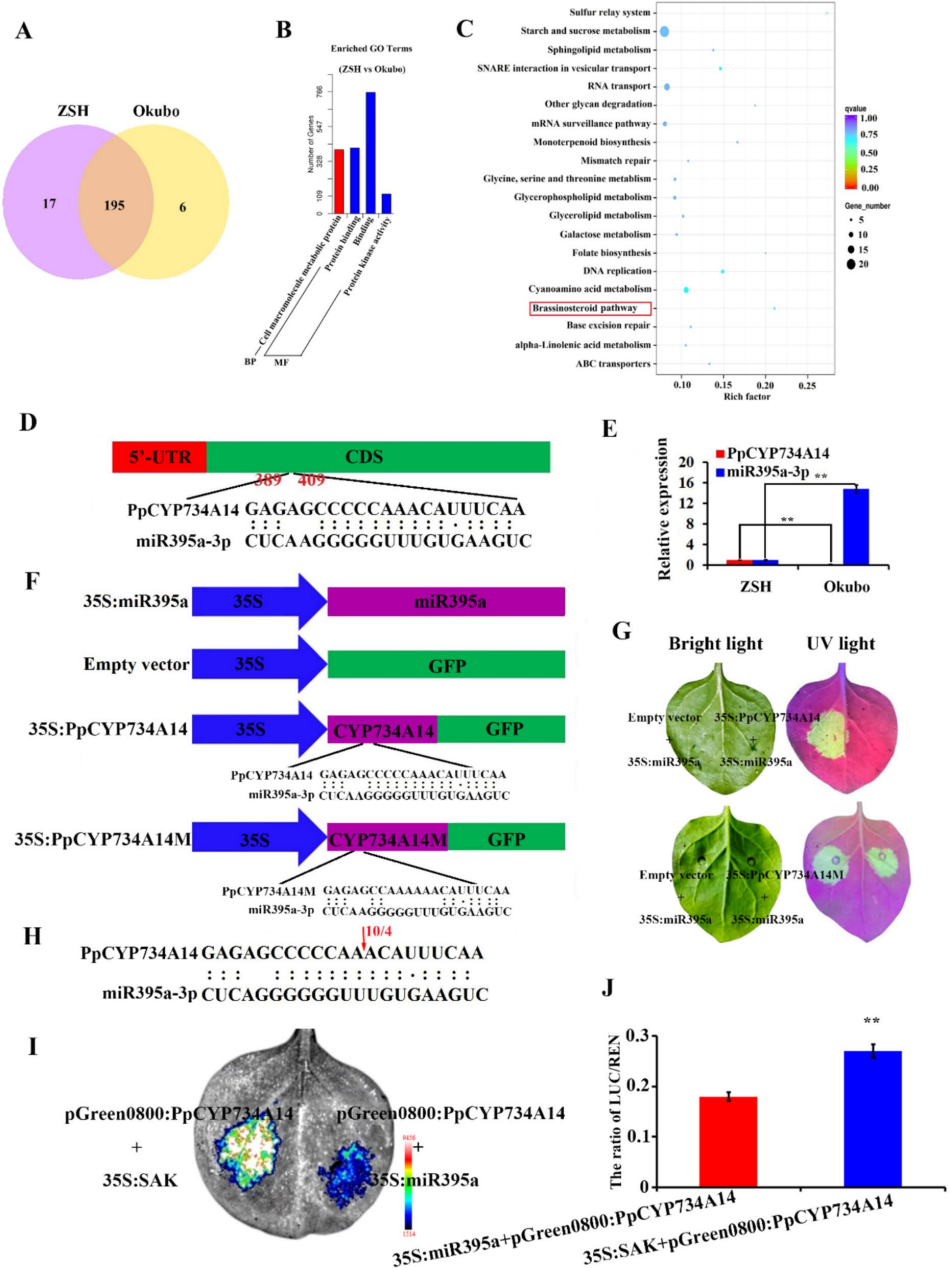
*PpCYP734A14* is a target of miR395a-3p. **A** Venn diagram illustrating DEMs between ZSH and Okubo. **B** GO enrichment analysis of DEM target genes (BP = biological process, MF = molecular function). **C** KEGG enrichment analysis of DEM target genes. **D** Base pairing relationship between *PpCYP734A14* and miR395a-3p. **E** Expression levels of *PpCYP734A14* and miR395a-3p in Okubo and ZSH. **F** Vectors utilized in transient expression assays in tobacco leaves. **G** Transient expression assays in tobacco leaves demonstrated that miR395a-3p targets *PpCYP734A14* by inhibiting GFP expression. **H** miR395a-3p cleavage site on *PpCYP734A14* was confirmed by 5'-RACE. **I**, **J** Dual-luciferase assays validating that miR395a-3p targets *PpCYP734A14*. Data are represented as mean ± standard deviation of three biological replicates. **p < 0.01 vs. control (Student's* t*-test)

To identify genes associated with peach branching, miRNA sequencing was performed on the axillary buds of the pillar peach ZSH and the standard peach Okubo, identifying 201 miRNAs in ZSH and 211 miRNAs in Okubo, with 195 miRNAs shared between these two cultivars (Fig. 2A). Compared to ZSH, 15 miRNAs were upregulated and six miRNAs were downregulated in Okubo (Wang et al. 2024). To explore the potential functions of these DEMs, their target genes were predicted. Gene Ontology (GO) analysis indicated that the target genes were primarily enriched in categories related to cellular macromolecule metabolic processes (BP), protein binding (MF), and protein kinase activity (MF) (Fig. 2B). KEGG pathway analysis revealed that the BR pathway contained four target genes of the DEMs (Fig. 2C).

Among the four BR-related target genes, only *PpCYP734A14*, a member of the CYP734As subfamily, exhibited significant differential expression and demonstrated substantially higher expression in ZSH than in Okubo (Fig. S1). Further analysis revealed that miR395a-3p, generated from the 3'-arm of pre-miR395a (Fig. S2), was involved in the BR catabolic pathway by targeting *PpCYP734A14*, indicating that differences in *PpCYP734A14* expression may contribute to the bioactive BR content variations between ZSH and Okubo.

To validate whether *PpCYP734A14* was the target gene of miR395a-3p, a prediction analysis of the miR395a-3p binding site was conducted, revealing that the target site is located within the coding sequence (CDS) region of *PpCYP734A14* (Fig. 2D). Expression analysis demonstrated an inverse correlation between miR395a-3p and *PpCYP734A14* in ZSH and Okubo, with miR395a-3p exhibiting significantly lower expression in ZSH, while *PpCYP734A14* showed higher expression in ZSH compared to Okubo (Fig. 2E). To confirm this interaction, the pre-miR395a sequence and the *PpCYP734A14* target site were cloned into pSAK277 and PMS4 vectors for transient expression assays (Fig. 2F). Co-transformation of PMS4 (35S:GFP) and 35S:miR395a in tobacco leaves produced strong GFP fluorescence, whereas the GFP fluorescence was absent when 35S:PpCYP734A14-GFP and 35S:miR395a were co-transformed, confirming that miR395a-3p targets *PpCYP734A14* and suppresses GFP expression (Fig. 2G). Upon mutation of the miR395a-3p binding site in *PpCYP734A14*, GFP fluorescence was restored, indicating interaction specificity. The cleavage site on *PpCYP734A14* was confirmed by 5'RLM-RACE, with results showing that four out of ten *PpCYP734A14* independent cDNA clones were cleaved between 9 and 10 nucleotides from the 5' end of miR395a-3p (Fig. 2H). Dual-luciferase assays further confirmed the targeting of *PpCYP734A14* by miR395a-3p (Fig. 2I, J). These findings demonstrated that miR395a-3p targets *PpCYP734A14* and reduces its mRNA levels.

**Fig. 3:**
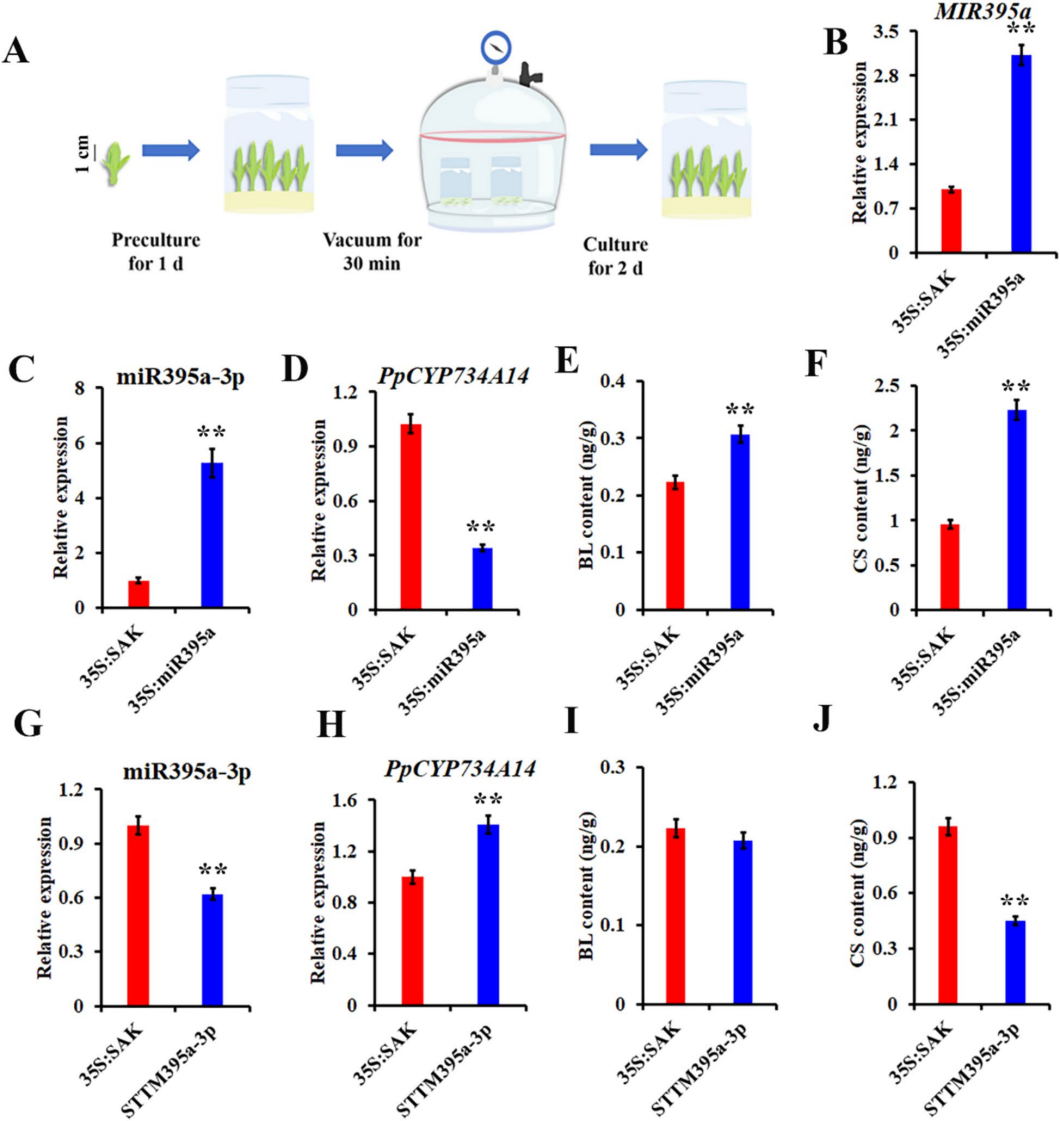
miR395a-3p inhibits *PpCYP734A14* expression and modulates BR metabolism. **A** Transient expression protocol in peach axillary buds. **B** Transient overexpression of pre-miR395a in peach axillary buds substantially increased miR395a-3p expression (**C**), which suppressed *PpCYP734A14* expression (**D**) and reduced endogenous bioactive BR content, including BL (**E**) and CS (**F**). Transient overexpression of STTM395a-3p inhibited miR395a-3p expression (**G**), resulting in elevated *PpCYP734A14* expression (**H**) and decreased bioactive BL (**I**) and CS (**J**) content. The empty vector 35S:pSAK277 served as a control. Values shown are the mean ± standard deviation of three biological replicates. **p < 0.01 vs. control (Student's* t*-test)

To further validate the role of miR395a-3p in regulating *PpCYP734A14*, transient overexpression and silencing experiments were conducted in peach axillary buds (Fig. 3A). Overexpression of pre-miR395a led to significantly elevated levels of mature miR395a-3p (Fig. 3B, C), a substantial reduction in *PpCYP734A14* expression (Fig. 3D), and a marked increase in bioactive BR content, including BL and CS (Fig. 3E, F). Conversely, transient silencing of miR395a-3p through STTM395a-3p resulted in reduced miR395a-3p expression (Fig. 3G), increased *PpCYP734A14* expression (Fig. 3H), and decreased bioactive BR content with a slight reduction in BL and significant decrease in CS (Fig. 3I, J). These results establish that miR395a-3p regulates bioactive BR content by targeting the BR catabolism gene *PpCYP734A14*.

### LncRNA7503 interacts with and destabilizes pre-miR395a

**Fig. 4:**
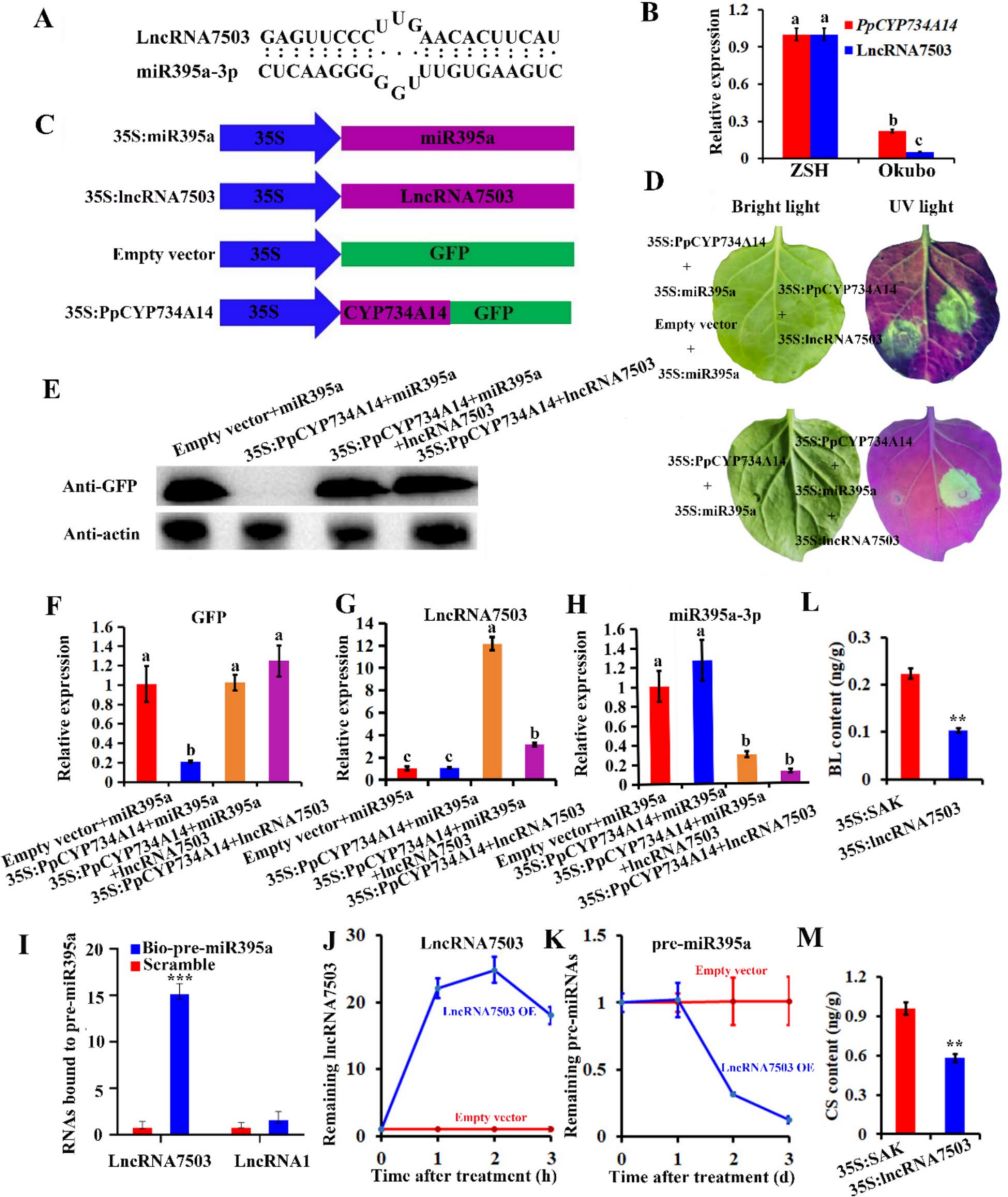
LncRNA7503 decreases miR395a-3p levels by binding to pre-miR395a. **A** Base pairing relationship between lncRNA7503 and miR395a-3p. **B** Expression of *PpCYP734A14* and lncRNA7503 in Okubo and ZSH. **C** Constructs employed in transient expression assays, driven by the 35S promoter. **D** Transient expression of PpCYP734A14-GFP with miR395a and lncRNA7503. **E** GFP protein levels in tobacco leaves by western blot, with β-actin as a loading control. **F** GFP expression in tobacco leaves by qRT-PCR. lncRNA7503 (**G**) and miR395a-3p (**H**) expression in agroinfiltration assays. GFP fluorescence and qRT-PCR were conducted in three biological replicates. **I** LncRNA7503 enrichment by biotin-labeled pre-miR395a; biotin-labeled scramble as control. LncRNA1 served as a control for specific binding. **J** Transient overexpression of lncRNA7503 significantly reduced pre-miR395a levels (**K**) and endogenous bioactive BRs BL (**L**) and CS (**M**). Values are the mean ± standard deviation; different lowercase letters indicate a significant difference between means (*p* < 0.05)

LncRNA sequencing was performed using axillary buds sampled from ZSH and Okubo (Wang et al. 2023). LncRNA, through binding to miRNA based on complementary sequences, can function as competing endogenous RNAs (*eTMs*) (Wu et al. 2013). To determine whether *eTMs* participated in peach branching formation, common mRNAs from miRNA-mRNA interaction pairs and lncRNA-mRNA co-expression pairs were analyzed (Fig. S3). Several transcripts (TCONS_00005387, TCONS_00005384, TCONS_00017503, TCONS_00090764, TCONS_00056547, and TCONS_00034025) were identified as potential *eTMs* of miR395a-3p (Fig. S3). Among these, the significantly differentially expressed transcripts TCONS_00017503 (lncRNA7503) and TCONS_00005384 (lncRNA5384), both displaying expression patterns similar to *PpCYP734A14* (Fig. S3), were selected for further investigation. The binding sites between miR395a-3p and lncRNA7503 (or lncRNA5384) were predicted through base pairing (Fig. 4A, Fig. S4A), and qRT-PCR analysis revealed that *PpCYP734A14*, lncRNA7503, and lncRNA5384 exhibited higher expression in ZSH compared to Okubo (Fig. 4B, Fig. S4B).

To validate the function of lncRNA7503 and lncRNA5384 as *eTMs* for miR395a-3p, pre-miR395a, lncRNA7503 (or lncRNA5384), and the *PpCYP734A14* binding site were cloned into pSAK277 and PMS4 vectors, respectively (Fig. 4C). Transient expression analyses showed that, compared to the control (empty + 35S:miR395a, 35S:lncRNA7503 + 35S:PpCYP734A14), GFP fluorescence was absent when 35S:PpCYP734A14 and 35S:miR395a were co-infiltrated. However, intense GFP fluorescence was detected when 35S:PpCYP734A14, 35S:miR395a and 35S:lncRNA7503 were co-infiltrated (Fig. 4D), suggesting that lncRNA7503 elevated *PpCYP734A14* mRNA levels. In contrast, co-infiltration with lncRNA5384 produced no GFP fluorescence (Fig. S4C), indicating that lncRNA5384 did not affect *PpCYP734A14* mRNA levels. Western blot and qRT-PCR analyses confirmed that lncRNA7503 enhanced *PpCYP734A14* expression by interfering with miR395a-mediated cleavage (Fig. 4E, F). Significantly, the lncRNA7503 mRNA level increased substantially when 35S:lncRNA7503 was co-infiltrated with 35S:miR395a + 35S:PpCYP734A14 or 35S:PpCYP734A14 (Fig. 4G), while miR395a-3p levels decreased significantly when lncRNA7503 was co-transferred with 35S:miR395a and 35S:PpCYP734A14 (Fig. 4H). Furthermore, 5'-RACE results indicated that lncRNA7503 was not cleaved by miR395a-3p.

RNA pull-down assays were conducted to examine the mechanism by which lncRNA7503 reduces miR395a-3p mRNA. The results demonstrated that lncRNA7503 was significantly enriched by biotin-labeled pre-miR395a, but not by biotin-labeled scramble oligomers, validating the specific interaction between pre-miR395a and lncRNA7503, as biotin-labeled pre-miR395a did not pull down lncRNA1 (Fig. 4I). Moreover, transient overexpression of 35S:lncRNA7503 in peach axillary buds (Fig. 4J) significantly enhanced pre-miR395a degradation compared to the control (Fig. 4K), leading to a substantial reduction in pre-miR395a half-life. Additionally, transient overexpression of lncRNA7503 significantly reduced the levels of bioactive BRs (BL and CS) in peach axillary buds (Fig. 4L, M). These results indicate that lncRNA7503 enhances *PpCYP734A14* expression by binding to and destabilizing pre-miR395a, thereby regulating bioactive BR content.

### *PpCYP734A14* and miR395a-3p affect peach branch number and angle

**Fig. 5:**
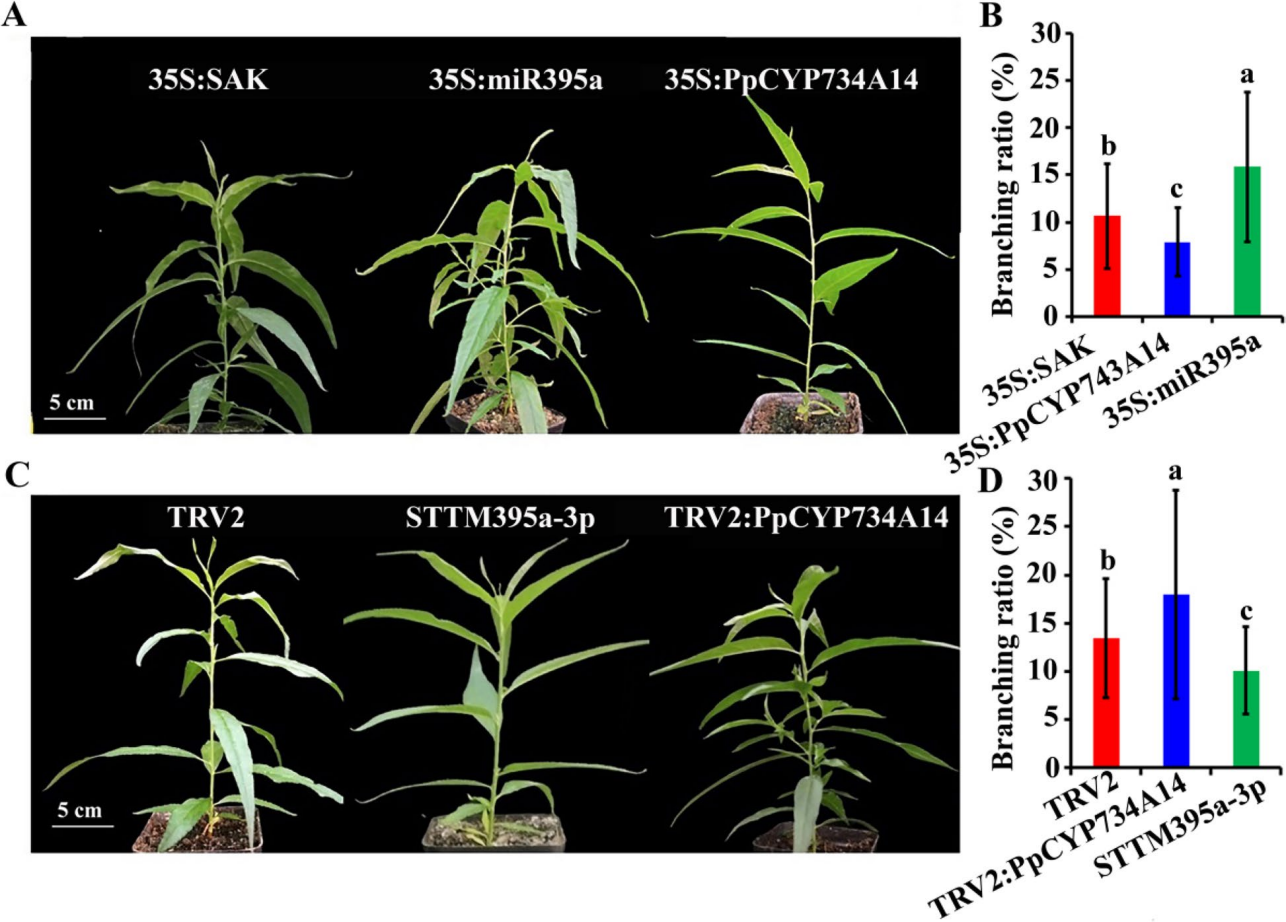
Branch number analysis of peach seedlings with altered *PpCYP734A14* and miR395a expression. Branch number (**A**) and branch ratio (**B**) in peach seedlings transiently overexpressing *PpCYP734A14* or miR395a. Branch number (**C**) and branching ratio (**D**) in seedlings with transiently silenced *PpCYP734A14* or miR395a. 35S:pSAK277, and TRV1 + TRV2 served as controls, respectively. Three biological replicates were performed. Values represent the mean ± standard deviation; different lowercase letters indicate a significant difference between means (*p* < 0.05)

To examine the functions of *PpCYP734A14* and miR395a-3p in regulating peach branch number and angle, overexpression and silencing constructs were generated and transiently expressed in peach seedlings. Seedlings overexpressing *PpCYP734A14* and miR395a-3p demonstrated significantly increased expression of both *PpCYP734A14* and miR395a-3p compared to the control group (Fig. S5, Table S1). The average branching ratios for the control, 35S:PpCYP734A14, and 35S:miR395a groups were 0.11, 0.08, and 0.16, respectively (Fig. 5A, B, Table S2). Conversely, when *PpCYP734A14* and miR395a-3p were transiently silenced (Fig. S4, Table S1), the branching ratios for the control, tumefaciens containing either TRV1 + TRV2 (TRV2:PpCYP734A14), and TRV2:miR395a were 0.13, 0.17, and 0.1, respectively (Fig. 5C, D, Table S2). These findings establish that *PpCYP734A14* significantly inhibits branching in peach, while miR395a-3p promotes branching.

**Fig. 6:**
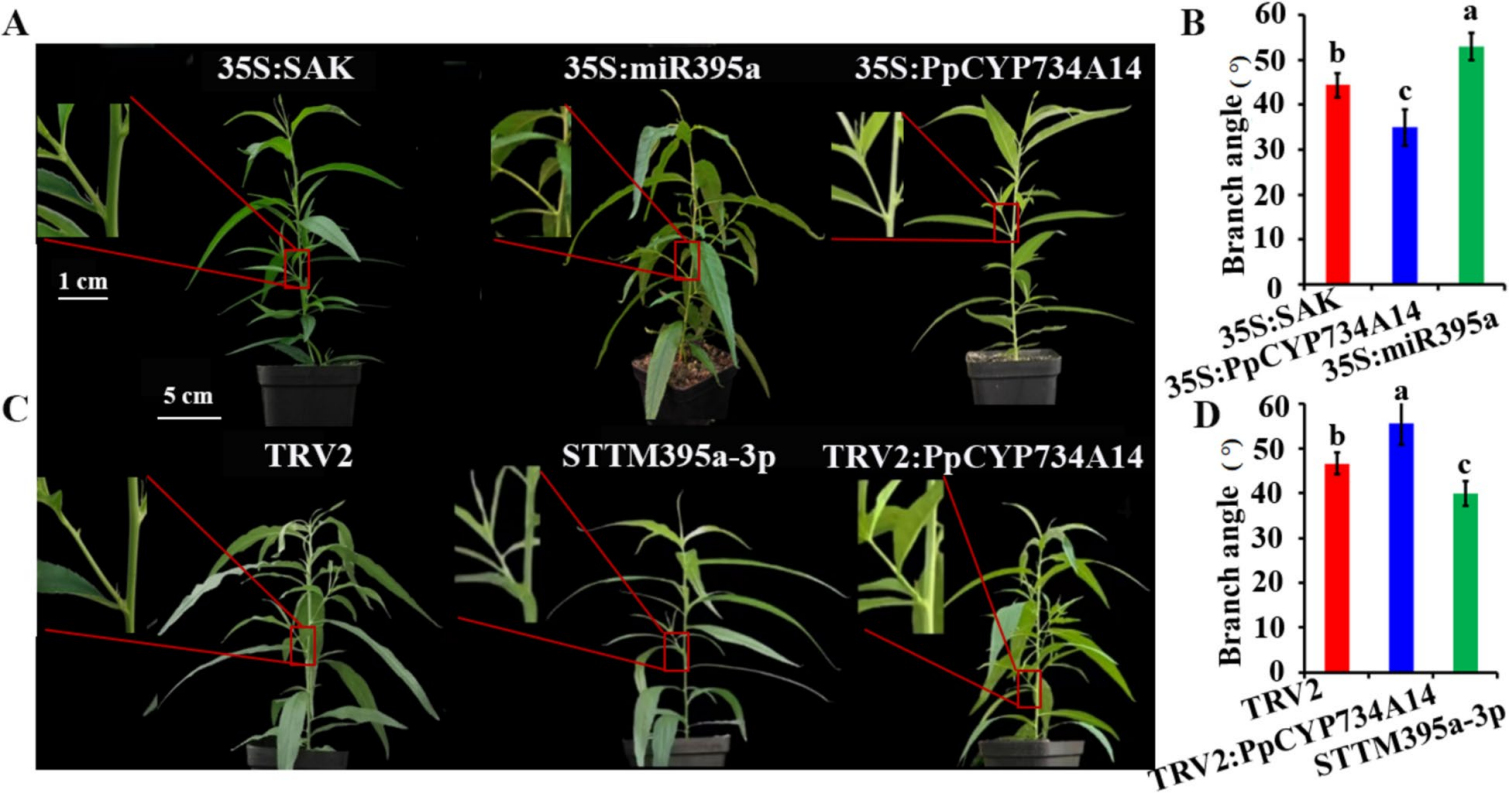
Branch angle analysis of peach seedlings with altered *PpCYP734A14* and miR395a expression. **A**, **B** Branch angle of peach seedlings transiently overexpressing *PpCYP734A14* and miR395a. **C**, **D** Branch angle of peach seedlings with transiently silenced *PpCYP734A14* and miR395a. Values are the mean ± standard deviation; different lowercase letters indicate a significant difference between means (*p* < 0.05)

The branch angle of peach seedlings with transient expression of *PpCYP734A14* and miR395a-3p was evaluated five weeks after transient expression. Relative to the control (35S:SAK), transient overexpression of *PpCYP734A14* significantly decreased branch angles, whereas overexpression of miR395a increased them (Fig. 6A, B, Table S3). The measured branch angles averaged 44.5°, 35°, and 53° for the control, 35S:PpCYP734A14, and 35S:miR395a groups, respectively. Conversely, silencing of *PpCYP734A14* and miR395a-3p yielded average branch angles of 47°, 55°, and 40° for TRV2, TRV2:PpCYP734A14, and STTM395a-3p, respectively (Fig. 6C, D, Table S3). These results indicate that *PpCYP734A14* substantially reduces branch angle in peach, while miR395a-3p increases it, presumably through targeting and downregulating *PpCYP734A14* mRNA.

## Discussion

Pillar peach trees demonstrate narrow canopy and necessitate less pruning due to reduced branch angles and fewer branches compared to standard peach trees (Bassi et al. 1994). Branching formation, a crucial determinant of tree architecture, has been thoroughly investigated in model plants (Kotov et al. 2021; Wang et al. 2022; Cao et al. 2022). In peach, previous research demonstrated that SL and BR both regulate branch number formation (Wang et al. 2024; Pei et al., 2025), with lncRNAs participating in this process by reducing miRNA levels. However, the differences in BR metabolism between pillar and standard peach cultivars remain unclear, as does the existence of genes affecting both branch number and angle.

### *PpCYP734A14* regulates peach branching through involvement in BR metabolism

The content of bioactive BRs is spatially and temporally regulated by their synthesis and catabolism due to the absence of long-distance BR transport. Consequently, bioactive BR inactivation serves a crucial role in maintaining the endogenous BR pool. The *CYP734A* family regulates bioactive BR content by converting active BRs into inactive forms (Sakamoto et al. 2011). Statistical analyses have revealed that pillar peach trees exhibit fewer branches (Wang et al. 2023) and smaller branch angles compared to standard peaches, with exogenous BR treatments significantly promoting branching (Wang et al. 2024) and markedly increasing branch angle. In this study, among the four identified BR-related genes targeted by DEMs, *PpCYP734A14*, associated with BR metabolism, demonstrated the highest expression in pillar peach trees. Transient overexpression of *PpCYP734A14* in peach axillary buds significantly reduced bioactive BL and CS content, while transient silencing of *PpCYP734A14* produced opposite results. These findings suggest that *PpCYP734A14* regulates endogenous bioactive BR content.

BRs play a crucial role in shoot architecture formation. In tomato, BR promotes bud outgrowth by inhibiting BRANCHED1 (*BRC1*) expression through the BR-responsive transcription factor BRASSINAZOLE-RESISTANT1 (BZR1) (Xia et al. 2021). In rice, tiller number increased through overexpression of BR synthesis genes *CYP724B* and *CYP90B* (Wu et al. 2008). Loss-of-function mutations in *CYP734A*, a BR metabolism gene, resulted in a BR deficiency phenotype characterized by dwarf plant height (Ohnishi et al. 2006; Sakamoto et al. 2011). In this study, transient overexpression or silencing of the BR metabolism gene *PpCYP734A14* affected both peach branch number and branch angle formation. *PpCYP734A14* likely influences branch number and angle through cell division and elongation regulation. Branch/tiller angles are determined by asymmetric growth at the branch/tiller base, with differences in cell length and number as the underlying cause (Roychoudhry and Kepinski 2015). Several BR biosynthesis genes regulate branch angle by affecting cell elongation (Azpiroz et al. 1998; Morinaka et al. 2006). These results indicate that *PpCYP734A14* influences peach branch number and angle through regulation of endogenous bioactive BR content.

### miR395a-3p expression alters peach branch number and angles

KEGG pathway analyses revealed that miRNA-targeted genes were enriched in starch, sucrose metabolism, and the BR pathway, a crucial hormone signaling process that promotes branching, indicating that miRNAs potentially regulate branching through involvement in the BR pathway. miRNAs serve essential functions in various plant development processes, including tree architecture, vegetative-to-reproductive transition, and responses to biotic and abiotic stresses (Zhai et al. 2020; Wang et al. 2021). For instance, miR172 is a conserved miRNA involved in ethylene and abscisic acid (ABA) pathways (Gao et al. 2012; Li et al. 2016), and miR395 regulates sulfate assimilation (Matthewman et al. 2012). In contrast to previous studies, our findings demonstrate that miR395a-3p targets the BR catabolism-related gene *PpCYP734A14*. miR395a-3p regulates bioactive BR content through interaction with and cleavage of *PpCYP734A14* mRNA. Transient overexpression of miR395a in peach seedlings produced more branches and larger branch angles, while transient silencing of miR395a-3p resulted in fewer branches and smaller branch angles. These findings suggest that miR395a-3p regulates branch number and angle by controlling bioactive BR content through interaction with *PpCYP734A14* mRNA.

### LncRNA7503 is involved in miR395a-3p-mediated peach branching by degrading pre-miR395a

LncRNA and miRNA interactions have been identified in plants. LncRNAs can inhibit miRNA-target interactions through sequence complementarity by binding to miRNA sequences (Yang et al. 2019). For example, the lncRNA *IPS1* inhibits *PHO2* cleavage by miR399 in *Arabidopsis* by functioning as a miRNA decoy (Franco-Zorrilla et al. 2007). TCONS_00021861 regulates rice drought tolerance by enhancing *YUCCA7* expression through miR528-3p sponging (Chen et al. 2021). Furthermore, *MLNC3.2* and *MLNC4.6* positively regulate the expression of *SPL2-like* and *SPL33* by acting as endogenous target mimics (*eTMs*) of miR156a (Yang et al. 2019), although the mechanism behind miR156a inhibition remains unclear. Similarly, our research indicates that lncRNA7503 enhances *PpCYP734A14* mRNA levels by decreasing miR395a-3p mRNA level. RNA pull-down assays and transient expression in peach axillary buds demonstrated that lncRNA7503 interacts with pre-miR395a and destabilizes it. Transient overexpression of lncRNA7503 in peach buds also significantly decreased bioactive BR content, indicating that lncRNA7503 reduces bioactive BR levels by promoting pre-miR395a degradation, thereby preventing *PpCYP734A14* cleavage by miR395a-3p.

### A model of bioactive BR content regulation by the lncR7503-miR395a-3p-CYP734A14 module affecting peach branch number and angle

**Fig. 7:**
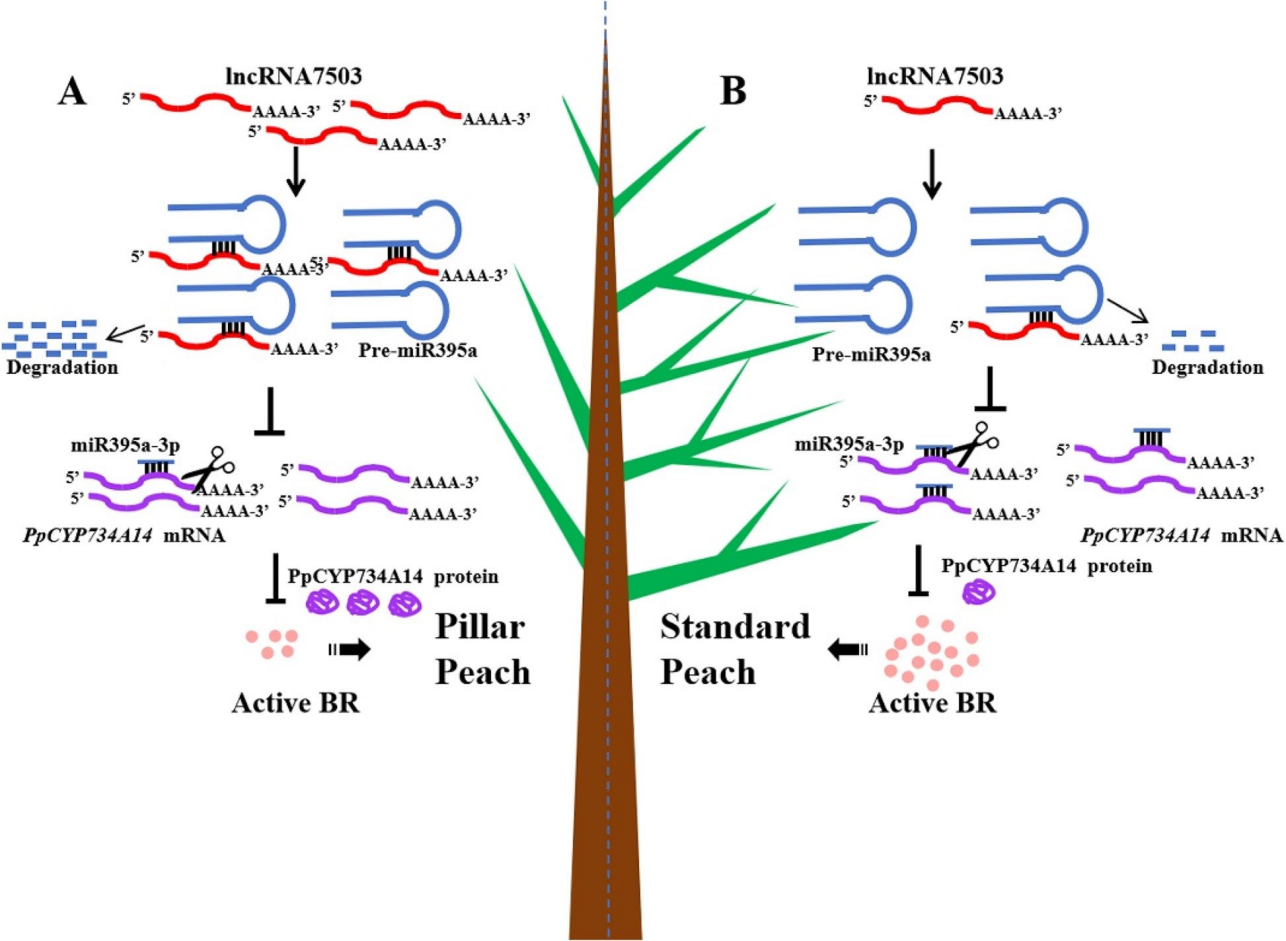
A proposed model illustrating the regulatory mechanism of lncRNA7503-miR395a-3p on peach branch architecture. **A** Elevated lncRNA7503 levels promote pre-miR395a degradation, resulting in enhanced *PpCYP734A14* expression, reduced bioactive BR content, and subsequent pillar peach morphology. **B** Decreased lncRNA7503 enables miR395a-3p to target and cleave *PpCYP734A14*, leading to elevated bioactive BR content and standard peach morphology

In summary, elevated levels of lncRNA7503 degrade pre-miR395a, reducing mature miR395a-3p levels. This reduction enables higher *PpCYP734A14* mRNA levels due to decreased miR395a-3p-mediated cleavage. Consequently, lower endogenous bioactive BR content suppresses peach branching and reduces branch angle, resulting in pillar peach trees with fewer branches and smaller angles (Fig. 7A). Conversely, low lncRNA7503 levels lead to reduced *PpCYP734A14* expression, increased bioactive BR content, and standard peach trees with more branches and larger branch angles (Fig. 7B).

## Methods

### Plant materials

Representatives of two peach tree types, designated as Okubo and ZSH, were cultivated in the research orchard of Henan Agricultural University, Zhengzhou, China. Axillary buds (pre-outgrowth) were collected from three-year-old Okubo and ZSH trees for miRNA and lncRNA sequencing. Axillary buds (post-outgrowth 2–3 cm) sampled from three-year-old ZSH trees were utilized for transient expression assays. One-year-old trees of cultivars Okubo and ZSH were specifically used for branch angle analysis. Exogenous BR was applied to one-year-old ZSH trees. *Nicotiana benthamiana* (tobacco) and wild peach MaoTao seedlings were used for transient expression and gene function analysis.

### Exogenous BR treatment

BR (10 mg·L^−1^) was applied to one-year-old ZSH trees through five spray applications, with three-day intervals between applications. Following treatment, the peach branch angles of the exogenous BR-treated group and control group were measured using the SC-K1 In-situ Live Plant Branch Angle Automatic Measurement System (China, Hangzhou, Wanshen). One-year-old ZSH trees treated with water served as the control group. The experiment included three biological replicates, with each replicate containing a minimum of five plants.

### miRNA/lncRNA analysis and targeted genes identification

miRNA and lncRNA libraries were constructed according to the methods described by Wang et al (2023, 2024). TargetFinder was employed to predict miRNA-targeted genes, which were subsequently analyzed using KEGG and GO databases through BLAST. The prediction of lncRNA-targeted genes was based on their co-location and co-expression with protein-coding genes.

### LncRNA-mRNA-miRNA networks construction

The identification of differentially expressed lncRNAs, miRNAs, and mRNAs was conducted based on the competing endogenous RNA (ceRNA) hypothesis. Cytoscape was utilized to visualize the lncRNA-miRNA-mRNA network.

### Quantitative real-time PCR (qRT-PCR) and gene cloning

TRIzol® reagent (Invitrogen) was used to extract total RNA from peach axillary buds. RNA purity assessment was performed using a 6000 Nano LabChip Kit (Agilent, CA, USA). RNA contamination and degradation were evaluated using 1% agarose gels, while RNA concentration was measured using a Nanodrop spectrophotometer. The RNA integrity assessment was conducted using the Agilent 2100 Bioanalyzer.

For miR395a-3p expression validation, total RNA was extracted from ZSH and Okubo using TRIzol®. The miRNA 1 st Strand cDNA Synthesis Kit (Vazyme) and Hiscript III RT SuperMix for qPCR (Vazyme) were used for cDNA synthesis. SYBR Select Master Mix (Thermo Fisher) was utilized for qRT-PCR on a LightCycler 96 (Applied BioSystems). U6 served as the normalization control for miRNA, while Actin was used for mRNA/lncRNA normalization. Primer Premier 5 was employed for specific primer design, and qRT-PCR was conducted following the protocol described by Zhai et al. (2020). The 2^−△△Ct^ method was applied to calculate expression levels for miRNA and lncRNA. *PpCYP734A14*, pre-miR395a, miR395a-3p, and lncRNA7503 were cloned according to Wang et al. (2023, 2024). Primers for qRT-PCR and gene cloning are listed in Table S4.

### Transient expression assay in tobacco leaves

Dual-luciferase (LUC) assays were conducted to verify miR395a-3p targeting of *PpCYP734A14*. The precursor of miR395a (Pre-miR395a) was inserted into the pSAK277 vector under the 35S promoter, while the CDS of *PpCYP734A14* was inserted into pGreenII-0800 vector. *Agrobacterium strains* containing 35S:pre-miR395a and pGreenII-0800:PpCYP734A14 were co-infiltrated into tobacco leaves. Empty vector 35S:pSAK277 served as control. LUC and REN activities were measured three days after infiltration.

To investigate the relationship between PpCYP734A14, miR395a-3p, and lncRNA7503, pre-miR395a and lncRNA7503 were inserted into pSAK277 vector, and the target site within *PpCYP734A14* CDS was inserted into PMS4 vector. *N. benthamiana* leaves were infiltrated using *A. tumefaciens*-mediated transient expression. Empty vector PMS4 served as control. GFP fluorescence was observed using a hand-held fluorescent lamp three days post-infiltration. Subsequently, total RNA and protein extraction was performed for qRT-PCR and Western blot analysis following Cai et al. (2014).

### Biotin-labeled RNA pull-down

LncRNA7503 was transiently expressed in peach axillary buds (after outgrowth). RNA extraction for RNA pull-down was performed three days later. Biotin-labeled pre-miR395a was bound to Streptavidin-Dynal beads and incubated overnight at 4℃ with rotation. After three washes, the beads were incubated for 6 h with RNA extracted from lncRNA7503 overexpression axillary buds. qRT-PCR analysis was performed on the bead-bound RNA. Scramble and lncRNA1 (Wang et al. 2024) served as controls.

### Transient expression in peach axillary buds

The conserved complementary sequence (approximately 200 bp) of *PpCYP734A14* was cloned into TRV2 to construct the *PpCYP734A14* silencing vector. For miR395a-3p, a suppression vector STTM was synthesized using miR395a-3p STTM sequence (listed in Table S4) following the protocol of Teotia & Tang (2017). The overexpression vector of PpCYP734A14, pre-miR395a, and lncRNA7503 was utilized for transient expression analysis using ZSH axillary buds (after outgrowth), as described by Wang et al. (2024). Specifically, peach axillary buds of ZSH were immersed in *A. tumefaciens* containing either TRV1 + TRV2 (TRV2:PpCYP734A14) or pSAK277 (pSAK277:PpCYP734A14, pSAK277:miR395a and pSAK277:lncRNA7503) and subjected to vacuum (approximately 0.8 Mpa) for 30 min, followed by cultivation for 2 days on MS medium. Subsequently, the peach axillary buds were frozen in liquid nitrogen for endogenous BR determination and qRT-PCR analysis, following Wang et al. (2024).

### Transient expression in peach seedlings

For gene function analysis, wild peach (MaoTao) seedlings were utilized for transient expression, following the method described by Cheng et al. (2024). The infected peach seedlings were transferred into soil and maintained under room temperature and normal light conditions. After approximately 1 month of cultivation, peach leaves were collected for qRT-PCR analysis, and the phenotype of transgenic lines was examined. Empty vectors pSAK277 and TRV1 + TRV2 served as negative controls. The experiment was performed with three biological replicates.

### Statistically analysis

All data were analyzed using SPSS software, with experiments conducted in triplicate. For pairwise and multiple comparisons, student's test (*, *P* < 0.05; **, *P* < 0.01) and Tukey's test (*P* < 0.05) were employed, respectively.

## Supplementary Information

Supplementary Material 1. Fig. S1. Expression of BR-related genes targeted by DEMs.

Supplementary Material 2. Fig. S2. The structure of pre-miR395a.

Supplementary Material 3. Fig. S3. LncRNA-miRNA-mRNA regulatory network.

Supplementary Material 4. Fig. S4. LncRNA5384 could not competitively bind to miR395a-3p.

Supplementary Material 5. Fig. S5. Transgenic lines identification.

Supplementary Material 6. Table S1. qRT-PCR data of peach seedlings transient expressed PpCYP734A14 and miR395a.

Supplementary Material 7. Table S2. Data (including internodes number and branch number) of peach seedlings transient expressed PpCYP734A14 and miR395a-3p (1 Month after culturing).

Supplementary Material 8. Table S3. Branch angle data with PpCYP734A14 and miR395a was transient overexpressed or silenced in peach seedlings.

Supplementary Material 9. Table S4 List of primers used in this study.

## Data Availability

The authors confirm that all data are included in the manuscript.

## Abbreviations

ZSH: ‘Zhaoshouhong’
BRs: Brassinosteroids
*TAC1*: TILLER ANGLE CONTROL 1
BL: Brassinolide
CS: Castasterone
*eTMs*: Endogenous Target Mimics
DEMs: Differential expression miRNAs
KEGG: Kyoto Encyclopedia of Genes and Genomes
GO: Gene Ontology
