# Supplementary figures and images for: LncRNA7503 decreases peach (Prunus persica) branch number and angle by inducing pre-miR395a degradation and reducing bioactive BR content
Source: Mol Hortic. 2026 May 7;6:31. doi: 10.1186/s43897-025-00215-6 (PMC13151148; doi:10.1186/s43897-025-00215-6)

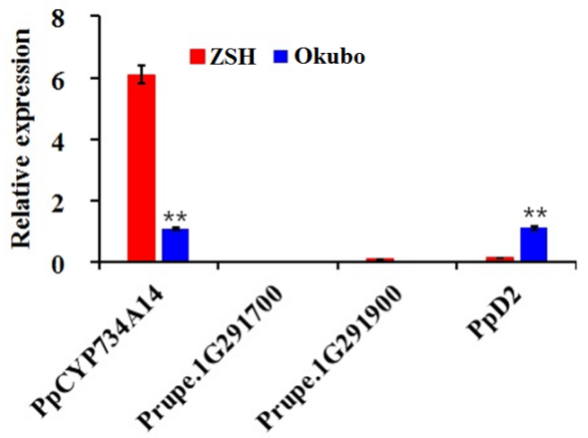


Fig. S1. Expression of BR-related genes in ZSH and Okubo targeted by DEMs.

Supplement: Supplementary file 1 — Supplementary Material 1. Fig. S1. Expression of BR-related genes targeted by DEMs. [file 43897_2025_215_MOESM1_ESM.docx]
