# Supplementary material for: LncRNA7503 decreases peach (Prunus persica) branch number and angle by inducing pre-miR395a degradation and reducing bioactive BR content
Source: Mol Hortic. 2026 May 7;6:31. doi: 10.1186/s43897-025-00215-6 (PMC13151148; doi:10.1186/s43897-025-00215-6)
Supplement: Supplementary file 2 — Supplementary Material 2. Fig. S2. The structure of pre-miR395a. [file 43897_2025_215_MOESM2_ESM.docx]

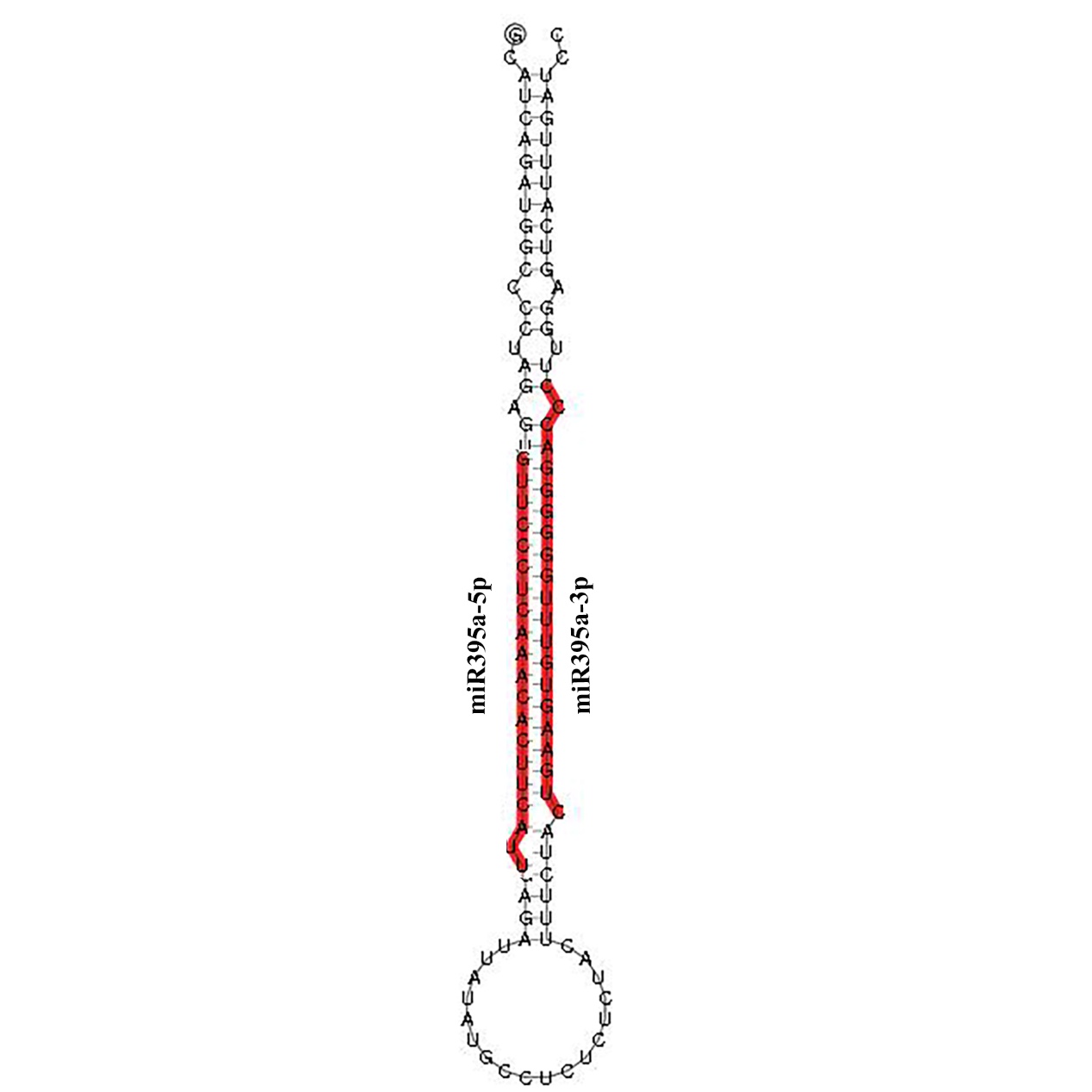


Fig. S2. The structure of pre-miR395a. The red highlighted sequence represent mature miR395a-3p and miR395a-5p generated from the 3'-arm and 5'-arm of pre-miR395a respectively.
