# Supplementary material for: LncRNA7503 decreases peach (Prunus persica) branch number and angle by inducing pre-miR395a degradation and reducing bioactive BR content
Source: Mol Hortic. 2026 May 7;6:31. doi: 10.1186/s43897-025-00215-6 (PMC13151148; doi:10.1186/s43897-025-00215-6)
Supplement: Supplementary file 3 — Supplementary Material 3. Fig. S3. LncRNA-miRNA-mRNA regulatory network. [file 43897_2025_215_MOESM3_ESM.docx]

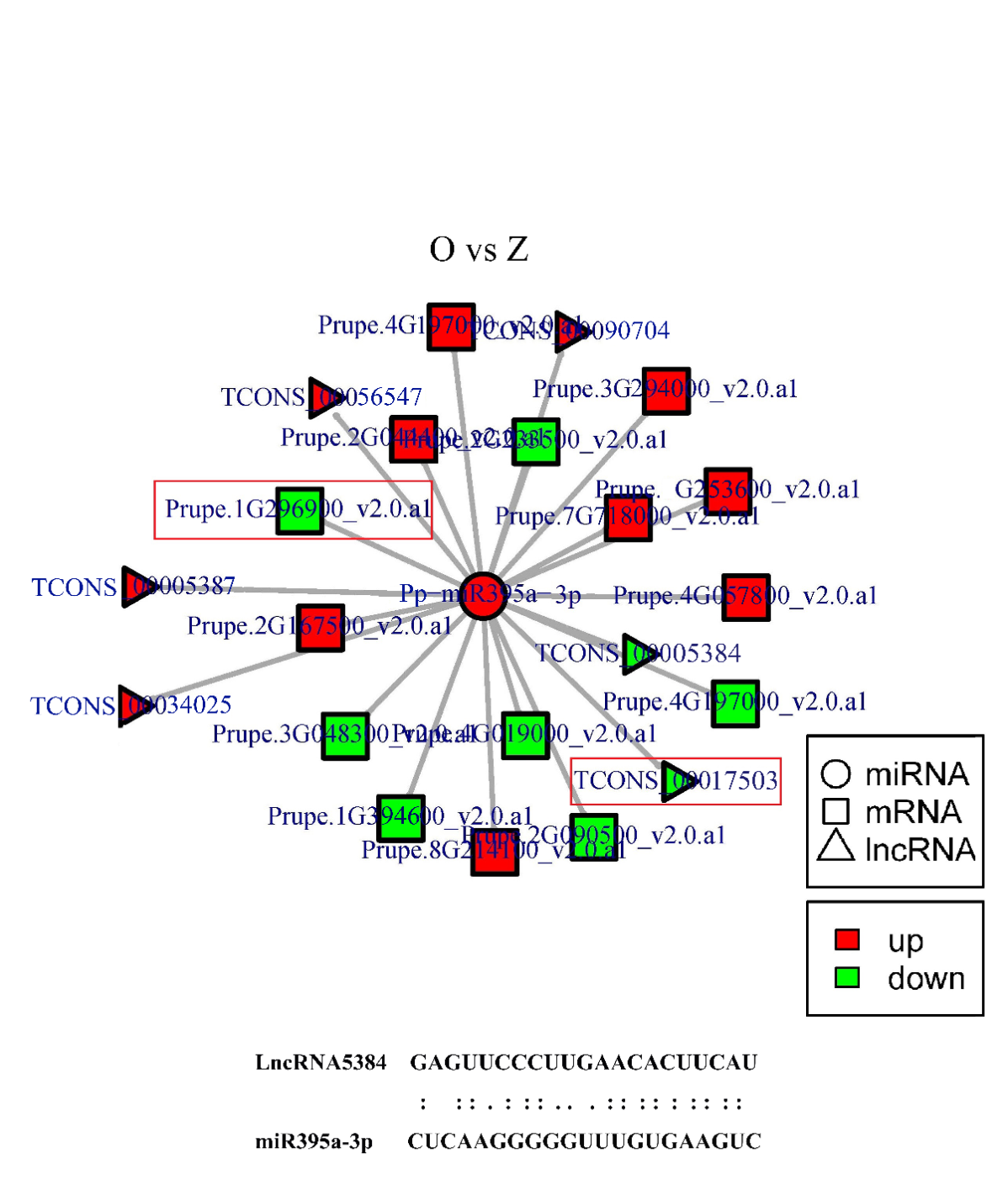


**Okubo vs ZSH**

Fig. S3. The LncRNA-miRNA-mRNA regulatory network. The red circles represented miR395a-3p. The red or green frame represented up- or down-regulated mRNA in Okubo compared with ZSH. Red and the green triangle represented down-regulated lncRNAs in Okubo compared to ZSH.
