# Supplementary material for: LncRNA7503 decreases peach (Prunus persica) branch number and angle by inducing pre-miR395a degradation and reducing bioactive BR content
Source: Mol Hortic. 2026 May 7;6:31. doi: 10.1186/s43897-025-00215-6 (PMC13151148; doi:10.1186/s43897-025-00215-6)
Supplement: Supplementary file 4 — Supplementary Material 4. Fig. S4. LncRNA5384 could not competitively bind to miR395a-3p. [file 43897_2025_215_MOESM4_ESM.docx]

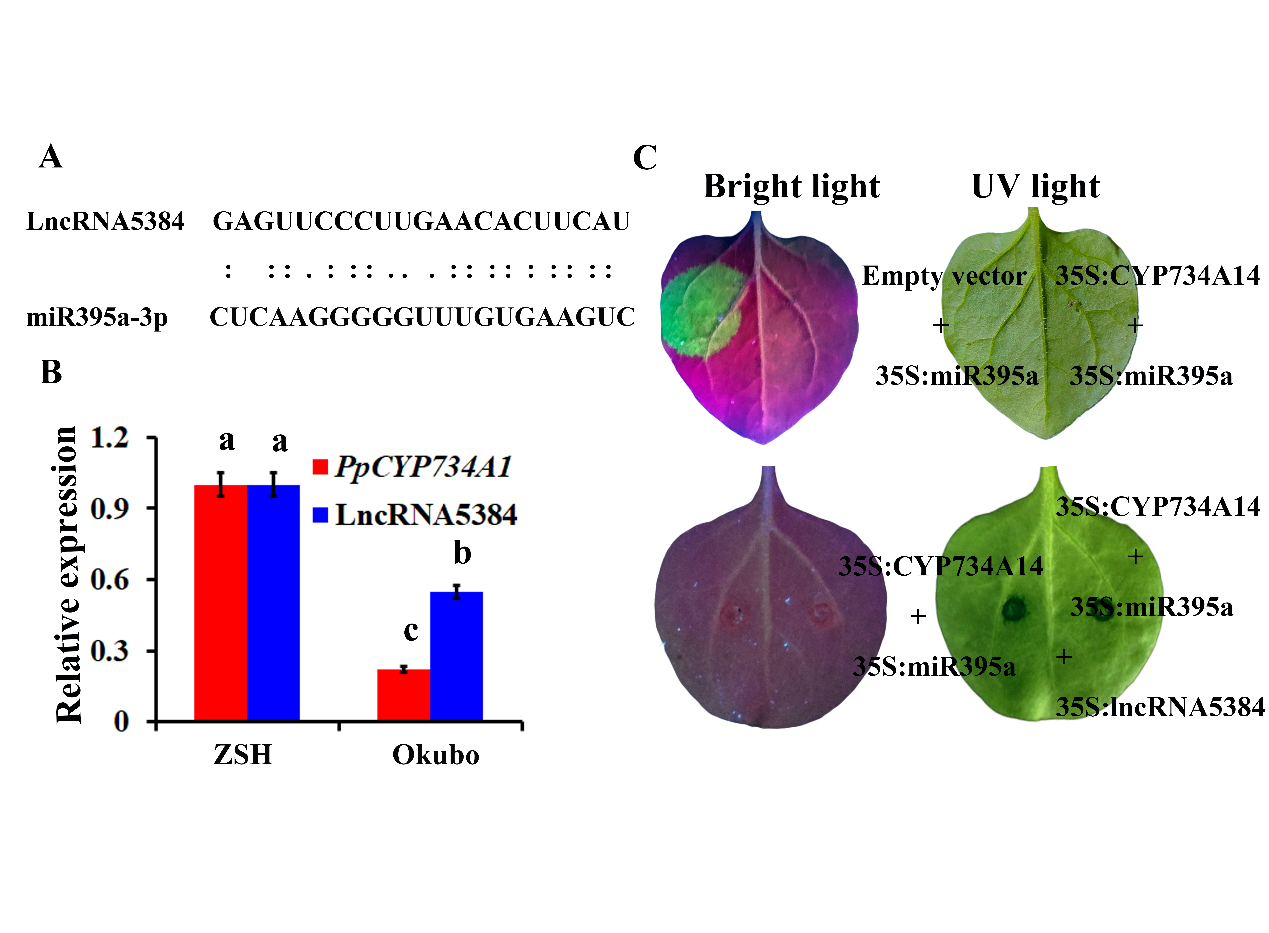


Fig. S4. LncRNA5384 could not competitively bind to miR395a-3p. **A** Base-pairing relationship between lncRNA5384 and miR395a-3p. **B** Expression of *PpCYP734A1* and lncRNA7503 in O and Z. **C** Transient expression of PpCYP734A1-GFP with miR395a and lncRNA5384. Values are the mean ± standard deviation; lowercase letters indicate a significant difference between means (*p* < 0.05).
