# Supplementary material for: LncRNA7503 decreases peach (Prunus persica) branch number and angle by inducing pre-miR395a degradation and reducing bioactive BR content
Source: Mol Hortic. 2026 May 7;6:31. doi: 10.1186/s43897-025-00215-6 (PMC13151148; doi:10.1186/s43897-025-00215-6)
Supplement: Supplementary file 5 — Supplementary Material 5. Fig. S5. Transgenic lines identification. [file 43897_2025_215_MOESM5_ESM.docx]

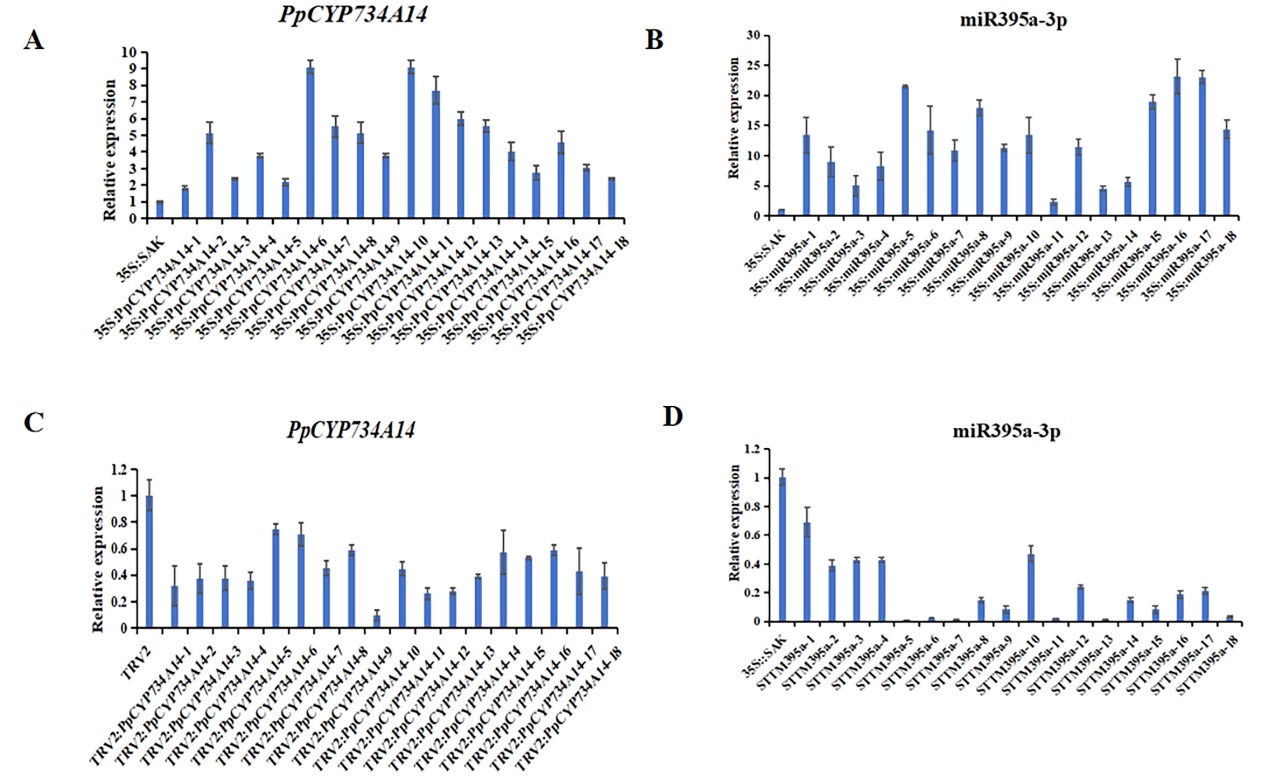


Fig. S5. Transgenic line identification. Expression level of *PpCYP734A14* (**A**) and miR395a-3p (**B**) transient overexpression in peach seedlings. The expression of *PpCYP734A14* (**C**) and miR395a-3p (**D**) in transient silenced peach seedlings.
